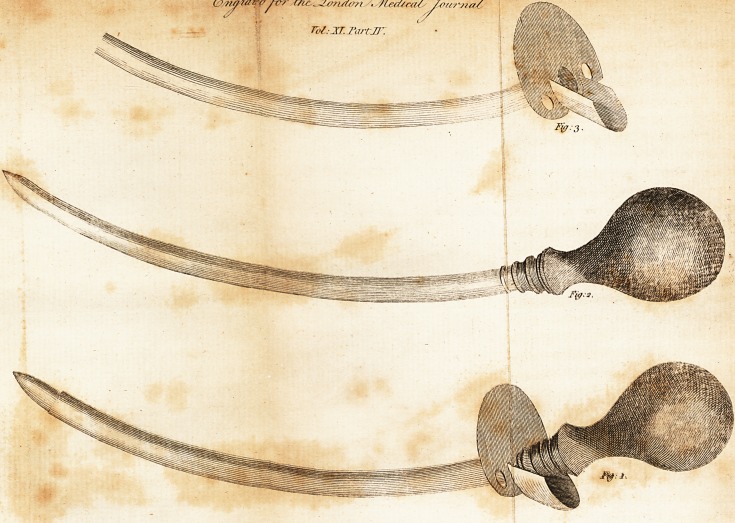# Some Reflections on the Paracentesis of the Urinary Bladder; with a Description of an Instrument Employed in Puncturing the Bladder through the Rectum

**Published:** 1790

**Authors:** Henry Watson

**Affiliations:** Surgeon to the Westminster Hospital


V. *Some Reflections on the Paracentesis of the urinary Bladder; with a Description of an Instrument employed in puncturing the Bladder through the Rectum. Communicated in a Letter to* Samuel Foart Simmons, *M. D. F. R. S. by Mr.* Henry Watson, *F. R. S. Surgeon to the Westminster Hospital.*

THE following reflections on the paracentesis of the bladder were suggested by a case of retention of urine, from external violence, to which I was called in consultation with Mr. William Norris, Surgeon, who has given an accurate account of it in the first volume of the Memoirs of the Medical Society. If they should appear to you worthy of attention, you will be pleased to communicate them to the Public through the medium of the London Medical Journal, a work of such extensive utility, that I am very sorry to find your other numerous avocations will not permit you any longer to continue it.

In the case alluded to, (the subject of which was a man thirty-six years old, who had received a severe blow on the perinæum in consequence of a fall from a scaffold) the difficulty in passing a catheter, catheter, or even the ſmalleſt bougie, ſeemed entirely owing to mechanical preſſure; for previouſly to the accident there was no diſeaſe in the parts concerned. The cellular membrane of the penis, ſcrotum, groins, and pubis, was loaded with extravaſated blood to a degree that gave great weight and uneaſineſs where it was lodged, and had very much the appearance of a gangrene, without being ſo.

In a ſimilar ſtate of the parts I ſhould think it very adviſable, firſt of all, to make pretty free inciſions on each ſide the ſcrotum, to unload the cellular membrane; for the preſſure being removed, it might then be poſſible to introduce a bougie, or even the catheter; though in the caſe in queſtion this could not have ſucceeded before the grumous blood was preſſed out. But when I was called, in conſultation with Mr. Norris and the other gentlemen who attended, the patient was ſuffering ſo cruelly from the over diſtention of his bladder, that I thought no time was to be loſt; therefore it was immediately determined that the bladder ſhould be punctured through the rectum, which operation was very carefully and well performed by Mr. Norris. The patient was immediately relieved, and his life preſerved.

In

In cafes of this fort, when the natural paffage is totally obftructed, there are more ways than one of getting into the urinary bladder; but the eafieft, fafeft, and moft advantageous operation fhould always be preferred.

Mr. Sharpe, in his Critical Inquiry, feems to think the puncture above the os pubis the moft eligible; but this, it is prefumed, cannot well be agreed to, becaufe it is an operation that muft certainly be attended with more than one inconvenience. This puncture is made through the fore part of the bladder, but not in the moft depending part; therefore a canula muft remain therein at leaft fo long as the natural paffage of the urethra is fhut up or obftructed.

The canula may produce inflammation: it has been known to make its way through the back part of the bladder, and even to have penetrated the rectum, and occafioned ulceration, fo that the urine has been evacuated with the fæces. Should the bladder recede from the canula, it will be almoft impoffible to replace it through the fame opening; in which cafe the urine may diffufe itfelf in the common connecting medium, and produce mifchief, much the fame as that which occafioned the high operation for the ftone to be fet afide. This mode is, is, therefore, not to be preferred, unlefs a dif-eafe within the rectum, or a diftempered prof-tate gland, fhould mark it out as the only eli-gible place—a place of neceffity, not of choice.

The puncture above the os pubis is certainly an eafy operation, requiring only a little cau-tion not to make the perforation too near the os pubis, or in the courfe of the epigaftric artery; and had it thofe advantages which are appa-rently wanting, it would certainly be the moft eligible mode of operating.

The puncture *in perinæo*, or what may be called the lateral pucture, is alfo recommended, but practifed by very few. It has, indeed, the advantage of making a depending opening; but the depth we have to penetrate, without a director, makes it, to many, a difagreeable operation, too much in the dark, requiring a critical knowledge of the anatomy of the parts: and fhould the obftruction, occafioning the re-tention of urine, originate in inflammation, ac-companied with any great tenfion, no man would be fond of plunging a canula through parts fo circumftanced.

The more fimple any operation can be ren-dered, the better it will be performed; the fewer fewer parts that are injured, the better fuccefs may always be expected from it.

Thefe confiderations may juftly induce us to give preference to the puncture through the rectum, provided the operation be performed in good time, before the bladder has loft its tone, and while the patient ftill poffeffes fome favourable fymptoms, otherwife no great advantage can be derived from it.

This operation, indeed, feems cleareft from objections of any that has been hitherto propofed; for if the gut and bladder are found, thefe, as parts to be wounded, are of no dangerous confequence: they are mufcular, and they are vafcular; the fize of the veffels renders them little liable to furnifh any confiderable hæmorrhage, and the mufcular fibres are always inclined to heal in good conftitutions. But to render the operation more certain, it is required that the bladder fhould be fufficiently diftended. I have known a patient bear the retention of urine not only many hours, but for feveral days, though this is by no means fafe. The diftention of the bladder comes on gradually, and is at firft very tolerable; but at length becomes fcarce fupportable: before this

 happens, happens, however, the patient ought to be relieved.

No parts of any great confequence are in the way of punċturing the bladder through the rectum, if we except the veficulæ feminales and the proftate gland: but if we reflect upon the fituation of thefe parts, we muft know that the vesicular bags divaricate in their afcent upon the bladder, and that it may be poffible to make the puncture between them without wounding either. The inftrument, to follow the right tract, muft be directed up the rectum, in a line exactly parallel to the fymphyfis of the offa pubis.

The proftate gland, if not difeafed, lies lower within the pelvis than we ought to perforate, and may therefore be confidered out of the queftion; though, fhould the veficulæ and proftate too be punctured, I think no very ill confequences are to be apprehended. I have known a portion of the veficulæ brought away by the forceps in cutting for the ftone, yet the patient did well; and we always conclude that the proftate gland is either torn or cut in the fame operation of lithotomy.

When the puncture has been made through the rectum, there can be no reafon for leaving a canula canula within the bladder any length of time, for the water will find a way through the depending perforation by its own gravity, and will continually paſs off as long as the urethra remains obſtructed. At firſt it always comes away with the fæces; yet no fæces will paſs from the rectum into the bladder, though theſe parts are ſo very contiguous, becauſe the wound is made in an oblique direction from below upwards; the force employed in expelling the ſtool is exerted in a contrary direction from above downwards; ſo that unleſs ſome great obſtruction occaſions an accumulation within the inteſtine, there can be little danger that even the thinner fæces ſhould regurgitate into the bladder.

Lately, as I have been informed, the puncture through the inteſtine has, in one caſe, been unſucceſsful; but this was confeſſedly owing to the operation having been attempted too late.

In a caſe related in the Medical Communications, Vol. I. page 256, the ſubject of which was but five years of age, and where the ſuppreſſion was of five days ſtanding, and attended with a gangrene of the neighbouring parts, with very fœtid urine, and every thing as unfavourable as could be, the operation ſucceeded perfectly, and the child recovered.

The inſtrument I employ for perforating the bladder through the rectum may be well underſtood from the annexed engraving, in which figure 2 repreſents the piercer; figure 3 the canula; and figure 1 the canula and piercer united. It conſiſts of two parts — a ſilver canula, and a piercer of poliſhed ſteel ſomewhat longer than the canula, terminating in a ſpear point like a lancet. Both canula and piercer have flat ſides, and are gently curved. They are to be introduced, in the united ſtate, as one inſtrument, the piercer being drawn a little within the canula ſo as to cover its point; two fingers of the right hand are then to be paſſed up the gut and along the groove formed between them: the inſtrument may be conducted gently, and made to preſs firmly againſt the bulging bladder; and the piercer, being then puſhed forwards, will enter the bladder, making an inciſed wound of about twice the ſize of an orifice in bleeding.

The ſilver canula is furniſhed with a ſpout, which, by directing the ſtream of urine properly, prevents its wetting the neighbouring parts. The piercer has a round wooden handle for the better ter holding and pufhing it forwards. The two inftruments fhould be nicely adapted to each other.

The pofition of the patient is to be the fame as in cutting for the ftone; but there is no neceffity for confinement by ligatures; neither are any dreffings needful after the operation.

*Rathbone Place,*

October 27, 1790.

## Figures and Tables

**Fig: 1. Fig: 2. Fig: 3. f1:**